# No clinically significant drug interactions between lenalidomide and P-glycoprotein substrates and inhibitors: results from controlled phase I studies in healthy volunteers

**DOI:** 10.1007/s00280-014-2438-4

**Published:** 2014-03-22

**Authors:** Nianhang. Chen, Daniel Weiss, Josephine Reyes, Liangang Liu, Claudia Kasserra, Xiaomin Wang, Simon Zhou, Gondi Kumar, Lilia Weiss, Maria Palmisano

**Affiliations:** Celgene Corporation, 86 Morris Avenue, Summit, NJ 07920 USA

**Keywords:** Lenalidomide, Digoxin, P-glycoprotein, Quinidine, Temsirolimus, Drug–drug interactions

## Abstract

**Purpose:**

Lenalidomide, a weak substrate of P-glycoprotein (P-gp) in vitro, is an oral anticancer drug eliminated predominantly via renal excretion as unchanged compound. The role of P-gp in lenalidomide disposition and the associated clinical relevance were evaluated.

**Methods:**

Two phase I, crossover studies were conducted in healthy volunteers. In Study 1, subjects received lenalidomide (10 mg × 7 days) alone or with the P-gp substrate digoxin (0.5 mg on Day 5). In Study 2, subjects received lenalidomide (a single 25 mg dose) alone, the P-gp inhibitor quinidine (300–600 mg twice-daily × 5 days) plus lenalidomide (on Day 4), the P-gp inhibitor/substrate temsirolimus (a single 25 mg dose) alone, or lenalidomide plus temsirolimus. Pharmacokinetic and safety data were collected for lenalidomide and the co-administrated drugs.

**Results:**

There were no significant changes in the maximum concentration (*C*_max_) and area under the plasma concentration–time curve (AUC) of lenalidomide when co-administered with quinidine, digoxin, or temsirolimus. Neither the rate nor the capacity of lenalidomide renal excretion was affected by quinidine or temsirolimus, in addition lenalidomide absorption rate and bioavailability remained unchanged. Furthermore, lenalidomide had no significant effect on blood *C*_max_ and AUC of temsirolimus and its active metabolite sirolimus (also a P-gp inhibitor/substrate). The *C*_max_ of digoxin was slightly higher (+14 %) when administered with lenalidomide versus placebo. There were no other changes in digoxin pharmacokinetics upon co-administration with lenalidomide. No remarkable safety findings were observed.

**Conclusions:**

There are no clinically significant pharmacokinetic interactions between lenalidomide and substrates or inhibitors of P-gp.

## Introduction

Lenalidomide is an oral IMiD^®^ immunomodulatory agent [1] with proven clinical efficacy and indicated in a range of hematological malignancies including: relapsed and/or refractory multiple myeloma (MM) [2, 3] in combination with dexamethasone; myelodysplastic syndromes associated with del(5q); [4, 5] and relapsed or refractory mantle cell lymphoma [6]. Lenalidomide has a predictable and manageable tolerability profile, with minimal neurotoxicity, allowing long-term administration [7]. Lenalidomide is rapidly absorbed (>90 %) after oral administration [8], and the disposition is similar in healthy subjects and patients [9]. Approximately 80 % of the administered lenalidomide dose is eliminated by renal excretion of the unchanged drug [10]. Clearance of lenalidomide is usually lower in patients due to compromised renal function resulting from the disease and advanced age.

P-glycoprotein (P-gp) is a 170 kDa transmembrane glycoprotein that functions as a biological barrier by extruding toxins and xenobiotics out of cells [11]. It is capable of transporting a wide array of structurally distinct compounds, including up to 50 % of currently marketed drugs [12]. P-gp is extensively expressed in the luminal membrane of the small intestine and blood–brain barrier, and in the apical membranes of excretory cells such as hepatocytes and the renal proximal tubule epithelia [13], hence contributing to the absorption, metabolism, distribution, and elimination of many drugs.

In monolayers of LLC-PK1 and MDCKII cell lines expressing human P-gp, lenalidomide is a weak substrate of P-gp, with an efflux ratio of approximately 3, compared with 18 for the prototypical P-gp substrate digoxin [14, 15]. In the P-gp expressing LLC-PK1 human cell line, lenalidomide up to a concentration of 300 μM did not inhibit P-gp-dependent transport of digoxin. Based on these in vitro findings, pharmacokinetic drug–drug interactions (DDI) between lenalidomide and substrates or inhibitors of P-gp are not anticipated to occur in vivo.

However, two recent publications have suggested anecdotal P-gp-mediated drug interactions in vivo, likely leading to increased lenalidomide exposure and toxicities. In an uncontrolled phase I dose-ranging study with MM patients [15], an increase in maximum drug concentration (*C*_max_) and the area under the concentration–time curve (AUC) of lenalidomide (25 mg/day) was observed with increasing doses of temsirolimus (15–20 mg/day), a known P-gp inhibitor/substrate. Also, an increase in *C*_max_ and AUC of temsirolimus (15 mg/day) was observed with increasing doses of lenalidomide (15–25 mg/day). Similarly, in a case report [16], the lenalidomide AUC was 12-fold higher in a MM patient receiving both lenalidomide (10 mg/day) and the P-gp inhibitor itraconazole (100 mg/day) compared with the AUC observed in other MM patients receiving lenalidomide (25 mg/day) alone. However, there was no difference in the elimination half-life (*t*_1/2_) of lenalidomide with or without itraconazole (5.5 vs. 6.7 h), which, in combination with the known high bioavailability of lenalidomide (>90 %), makes it difficult to account for a 12-fold increase in AUC. Furthermore, these two studies are confounded with lack of control groups, small sample size, and use of multiple co-medications.

Nevertheless, the above reports raised an important clinical question: What is the “true” nature of the supposedly P-gp-mediated DDI of lenalidomide in spite of the low potential of such interaction based on in vitro findings and would it lead to increased toxicity? In vitro data have been found to underestimate the in vivo effects of some drugs on pharmacokinetics of P-gp substrates [12]. Hence, we conducted prospective, well-controlled clinical studies to evaluate the effect of three P-gp probe drugs on the pharmacokinetics of lenalidomide, and the effect of lenalidomide on the pharmacokinetics of three P-gp probe drugs. The three probe drugs are as follows: the prototypical P-gp substrate digoxin, the well-characterized strong P-gp inhibitor quinidine, and the P-gp inhibitor/substrate temsirolimus, which was previously suggested to have pharmacokinetic interactions with lenalidomide [15].

## Subjects and methods

### Study population

Subjects were healthy male or female volunteers with a body mass index of 18–33 kg/m [2], aged between 18 and 65 years, and in good health as determined by medical history, physical examination, and clinical laboratory tests; as well as negative for hepatitis, HIV, and no history of drug or alcohol abuse. Subjects who were treated with Pg-p inhibitor/inducers 30 days before first dose administration were excluded from enrollment. Other exclusion criteria included the use of any prescribed or non-prescribed systemic or topical medication (including vitamin/mineral supplements and herbal medicines). Permitted concomitant medications included paracetamol, doxycycline, and bactrim, and any medication necessary to treat adverse events (AEs) or medical emergencies. The studies were conducted in accordance with principles of Good Clinical Practice and were approved by the appropriate institutional review boards in accordance with the Declaration of Helsinki. All subjects provided written informed consent prior to initiation of study.

### Study design and treatment

Two phase I, single-center studies were conducted to evaluate the effect of digoxin (Study 1), quinidine (Study 2, Part 1), and temsirolimus (Study 2, Part 2).

#### Evaluation of lenalidomide interaction with digoxin

Study 1 was designed as a double-blind, placebo-controlled, randomized, 2-period, crossover trial. Subjects were randomized to receive either lenalidomide 10 mg/day for 7 days in Study Period 1 and the matching placebo for 7 days in Study Period 2, or to receive the same two treatments in the reverse order. On Day 5 of both periods, a single oral 0.5 mg dose of digoxin (2 × 0.25 mg) was administered immediately after oral ingestion of lenalidomide. A 10-day washout separated the study periods. On Day 4 of both periods, serial blood samples up to 24 h were collected for measurement of lenalidomide plasma concentrations. Following administration of digoxin, a 72-h blood sample was collected to determine the plasma concentrations of both digoxin and lenalidomide.

#### Evaluations of lenalidomide interaction with quinidine

Study 2, Part 1 was designed as an open-label, fixed-sequence, 2-period, crossover trial. In Period 1, subjects received a single oral dose of 25 mg lenalidomide. In Period 2, subjects received 300 mg quinidine sulfate (extended-release tablets) twice-daily on Day 1 and 600 mg twice daily on Days 2–5, with a single 25 mg lenalidomide dose immediately following the morning quinidine dose on Day 4. Study periods were separated by a washout of 7–10 days. In both periods, serial blood samples were collected, and urine samples were pooled over specified intervals for up to 48-h post-lenalidomide dosing to determine plasma and urine lenalidomide concentrations. In Period 2, serial blood samples were collected prior to each morning quinidine dose, and at specified times for 12-h post-morning quinidine dose on Day 4 to determine the plasma quinidine concentrations.

#### Evaluation of lenalidomide interactions with temsirolimus

Study 2, Part 2 was designed as an open-label, fixed-sequence, 3-period, crossover trial. In Period 1, subjects received a single oral dose of 25 mg lenalidomide; in Period 2, subjects received a single dose of temsirolimus 25 mg via a 30-min intravenous (IV) infusion; and in Period 3, subjects received both a single 25 mg oral dose of lenalidomide and a single 25 mg IV dose of temsirolimus. Pre-treatment with IV diphenhydramine (25 mg) was administered 30 min prior to start of each temsirolimus infusion. There was a 7–10 day washout between Study Periods 1 and 2, and a minimum 14-day washout between Study Periods 2 and 3. In Periods 1 and 3, serial blood samples were collected, and urine was pooled over specified intervals for up to 48-h post-lenalidomide dosing to determine plasma and urine lenalidomide concentrations. In Study Periods 2 and 3, serial blood samples were collected for 168-h after start of temsirolimus infusion to determine concentrations of temsirolimus and its active metabolite sirolimus in whole blood.

### Bioanalytical methodology

Concentrations of lenalidomide, quinidine, temsirolimus, and sirolimus in biological fluids were determined by validated liquid chromatography mass spectrometry assays. For analysis of lenalidomide in plasma and urine, the assay ranges were 5–1,000 and 100–20,000 ng/mL, respectively, and the details of methods were described previously [8, 10]. For the analysis of quinidine in plasma, the assay range was 20–20,000 ng/mL. Quinidine and its internal standard, quinidine-*d3*, were quantitatively extracted from each plasma sample by protein precipitation extraction. For the analysis of temsirolimus and its active metabolite sirolimus in whole blood, the assay range was, respectively, 0.25–125 and 0.1–50 ng/mL. Sirolimus, temsirolimus, tacrolimus, and [[13] C [2, 3] H7]-temsirolimus were quantitatively extracted from whole blood sample by protein precipitation extraction. Concentrations of digoxin in plasma were determined by a validated radioimmunoassay method using the Becton Dickinson Digoxin Solid-Phase Component System. The method allowed the direct measurement of digoxin using a digoxin-specific antibody immobilized on the wall of a polypropylene tube, and the assay linear range was 0.1–50 ng/mL.

### Pharmacokinetic analysis

Non-compartmental pharmacokinetic parameters were estimated from drug concentration–time profiles. Major pharmacokinetic parameters included *C*_max_, time to reach *C*_max_ (*T*_max_), terminal-phase half-life (*t*_1/2_), AUC from time zero to the last measurable concentration (AUC_t_), AUC from time zero to infinity (AUC_∞_), cumulative urinary excretion as a percentage of administered dose (fe), and renal clearance (CL_R_).

### Statistical analysis

An analysis of variance (ANOVA) was performed on the natural log-transformed pharmacokinetic parameters to estimate the percentage ratio of geometric means between treatments, and the 90 % confidence interval (CI) for key pharmacokinetic parameters. The ANOVA model included sequence, treatment, day, and period as fixed effects and subject nested within sequence as a random effect for comparison of a digoxin pharmacokinetic parameter with and without lenalidomide. For evaluation of the interaction between lenalidomide and quinidine or temsirolimus, the ANOVA model included treatment as a fixed effect and subject as a random effect for comparison of a drug pharmacokinetic parameter with and without the interacting drug.

### Safety analysis

Safety was evaluated throughout each study by monitoring AEs, physical examinations, vital signs, clinical laboratory values, and 12-lead electrocardiogram results and was summarized using descriptive statistics.

## Results

### Study population

A total of 50 healthy subjects were enrolled. All subjects had renal and hepatic functions that were within normal institutional limits.

In Study 1, evaluation of lenalidomide interactions with digoxin, 19 healthy subjects (five females and 14 males) were randomized with 17 subjects completing the study. Of these subjects, 15 (79 %) were African American and 4 (21 %) were white. The mean age was 37 years (range: 22–48 years). One subject discontinued the study at the discretion of the investigator for non-compliance and one subject voluntarily withdrew consent for study participation.

In Study 2, Part 1, evaluation of lenalidomide interactions with quinidine, 14 healthy male subjects were enrolled, and all subjects completed the study. Of these subjects, 9 (64.3 %) were African American, and 5 (35.7 %) were white. The mean age was 38 years (range: 26–62 years).

In Study 2, Part 2 (lenalidomide interactions with temsirolimus) 17 healthy male subjects were enrolled, and 11 completed the study. Of these subjects, 10 (58.8 %) were African American, and 7 (41.2 %) were white. The mean age was 37 years (range: 22–56 years). In total 6 subjects did not completed the study, 4 voluntarily withdrew consent for study participation, 1 was lost to follow-up after Period 1, and 1 was withdrawn due to an AE of furuncle on Day 27 of Period 2.

### Effect of co-administered drugs on lenalidomide pharmacokinetics in plasma

**Fig. 1:**
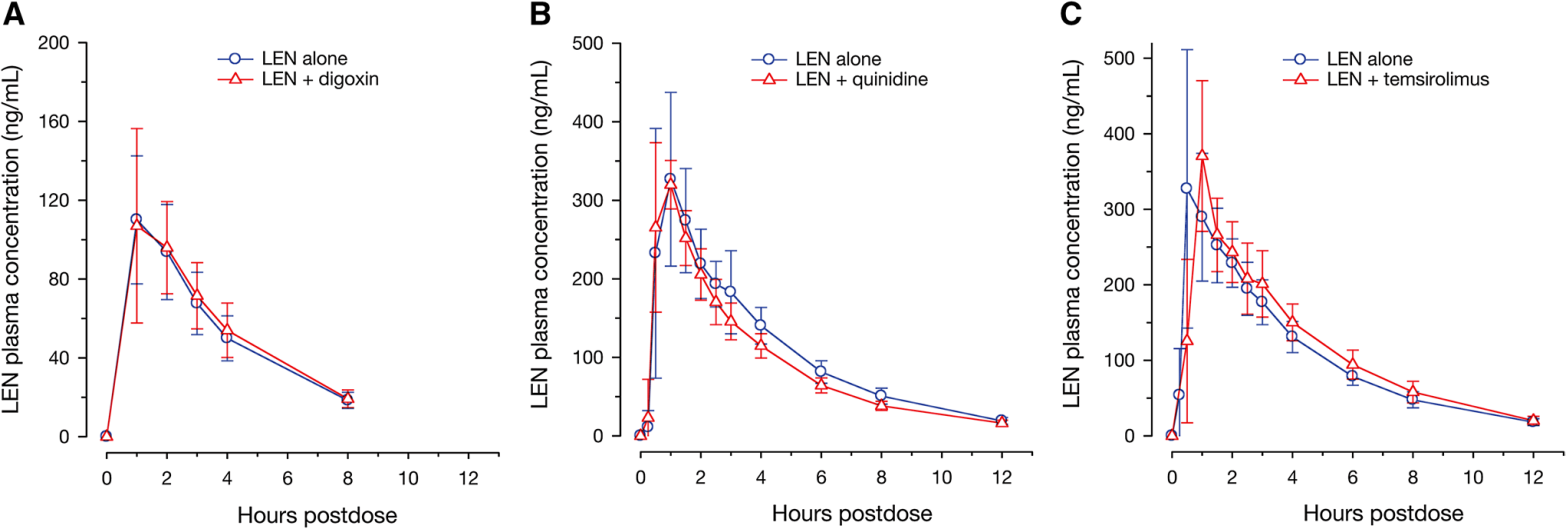
Mean (±standard deviation) plasma concentration–time profile of lenalidomide alone and in the presence of (**a**) digoxin, (**b**) quinidine, or (**c**) temsirolimus in healthy subjects

**Fig. 2:**
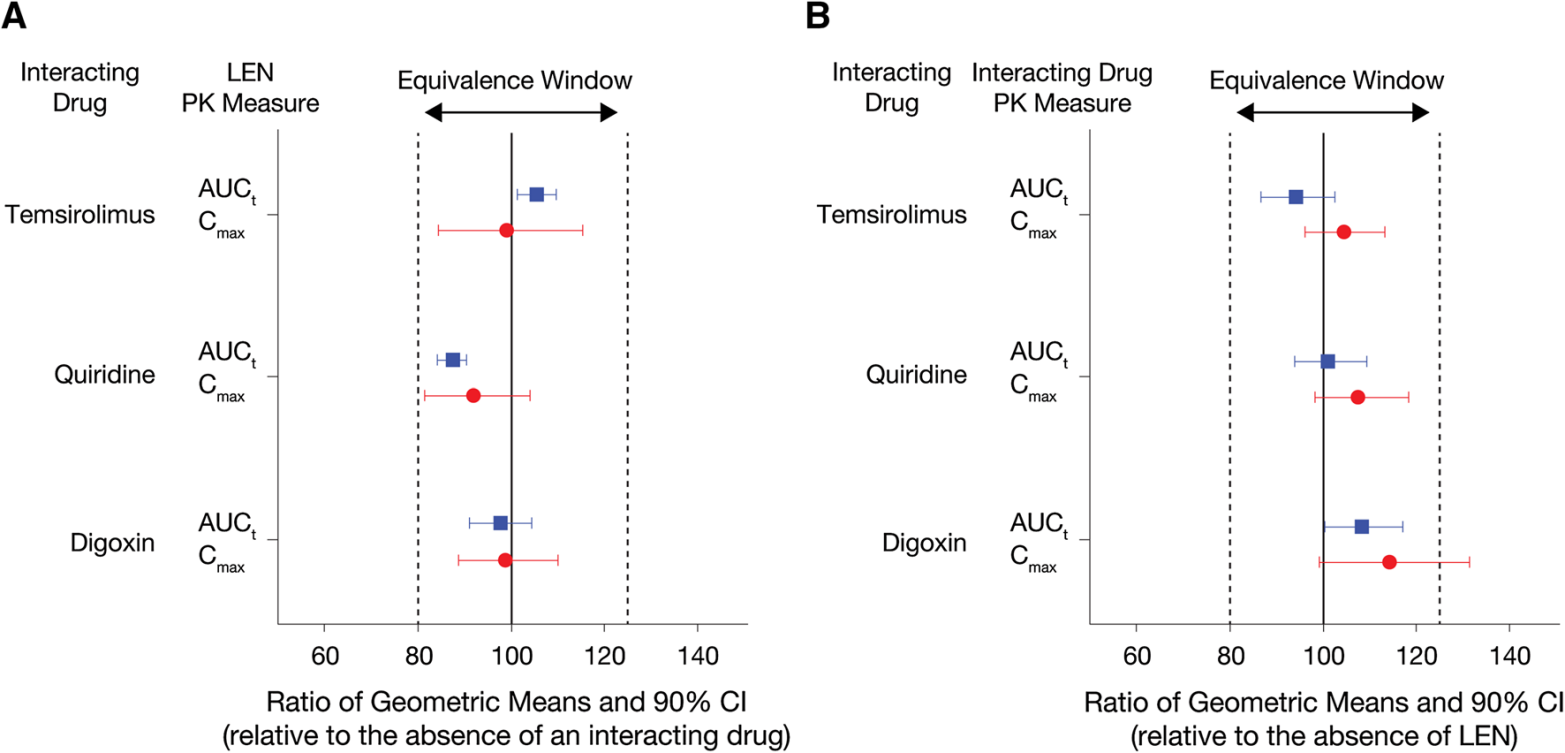
Ratio of geometric means and associated 90 % confidence interval for systemic exposure (*C*_max_ and AUC_t_) of lenalidomide (**a**) and interacting drugs (**b**) when co-administered

The time profiles of mean plasma concentrations for lenalidomide were similar when administered alone or in combination with digoxin (Fig. 1a), quinidine (Fig. 1b), or temsirolimus (Fig. 1c). Key pharmacokinetic parameters of lenalidomide are summarized in Table 1. There was no difference in the rate of lenalidomide absorption when administered alone or in combination with any one of the interacting drugs; the observed *C*_max_ was similar and occurred at a median time of 0.5–1 h under all conditions. After reaching *C*_max_, lenalidomide concentrations declined in a similar pattern regardless of treatment. No differences were observed in the mean *t*_1/2_ between treatments. In statistical comparisons, the 90 % CIs for the ratio of geometric means between lenalidomide alone, and lenalidomide plus an interacting drug were completely contained within the limits of 80 and 125 % for *C*_max_ and AUC of lenalidomide in plasma (Fig. 2a).

**Table 1:** Pharmacokinetic parameters of lenalidomide alone and in the presence of digoxin, quinidine, or temsirolimus

Pharmacokinetic parameter	Lenalidomide (10 mg)		Lenalidomide (25 mg)		Lenalidomide (25 mg)	
	Alone (*n* = 17)	+Digoxin (*n* = 17)	Alone (*n* = 14)	+Quinidine (*n* = 14)	Alone (*n* = 17)	+Temsirolimus (*n* = 11)
AUC_t_ (h·ng/mL)	396 (32.7)	386 (38.9)	1,288 (12.1)	1,127 (9.6)	1,276 (12.0)	1,366 (14.5)
AUC_∞_ (h ng/mL)	475 (23.2)	491 (22.2)	1,361 (12.7)	1,190 (9.8)	1,351 (11.9)	1,445 (14.5)
C_max_ (ng/mL)	119 (20.2)	118 (32.8)	367 (26.3)	337 (12.3)	364 (30.0)	361 (24.8)
T_max_ (h)	1 (1–2)	1 (1–2)	1 (0.5–3)	1 (0.5–1.5)	0.5 (0.5–2)	1 (1–2)
t_1/2_ (h)	2.40 (21.0)	2.41 (17.0)	2.81 (10.1)	2.86 (12.9)	2.81 (10.5)	2.69 (9.1)
CL_R_ (mL/min)	ND	ND	227 (18.3)	245 (11.3)	251 (16.4)	229 (15.6)
fe (% dose)	ND	ND	74.2 (11.4)	70.2 (6.6)	81.0 (10.0)	79.6 (8.0)

Geometric mean (geometric CV %) data are presented for all parameters except for *T*_max_ where median (range) data are presented

*AUC* area under the plasma concentration curve, *AUC*_*t*_, AUC from time zero to the last measurable concentration, *AUC*_*∞*_ AUC from time zero to infinity, *CL*_*R*_ renal clearance, *C*_*max*_ maximum observed plasma concentration; fe, cumulative urinary excretion as a percentage of administered dose, *ND* not determined, *t*_*1/2*_ z terminal-phase half-life, *T*_*max*_ time to reach *C*_max_

### Effect of co-administered drugs on lenalidomide pharmacokinetics in urine

**Fig. 3:**
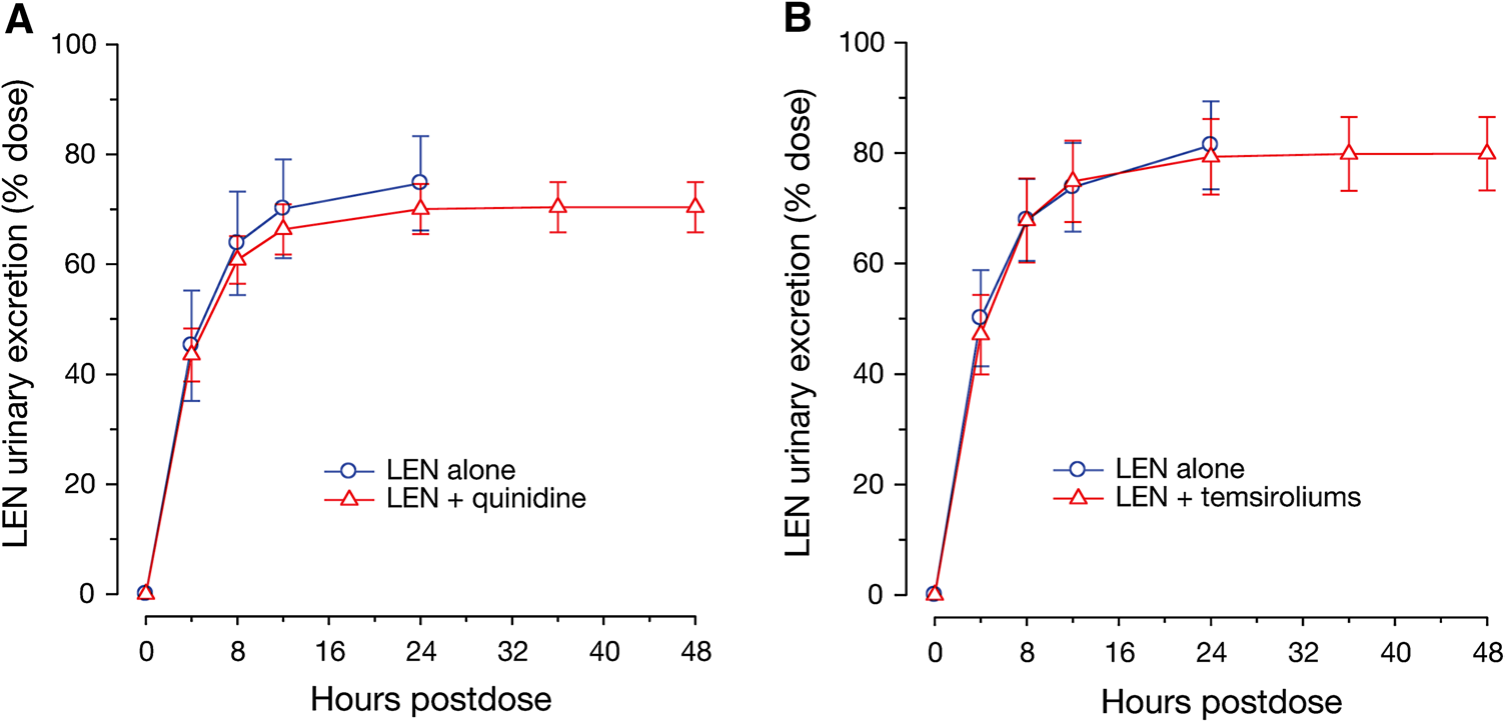
Mean (±standard deviation) renal excretion-time profile of lenalidomide alone and in the presence of (**a**) quinidine or (**b**) temsirolimus in healthy subjects

Effect of P-gp inhibition on renal excretion of lenalidomide was evaluated using quinidine and temsirolimus. Under all treatment conditions, the urinary excretion of unchanged lenalidomide was nearly complete by 12-h post-dose (Fig. 3). The mean renal clearance and mean percentage of unchanged lenalidomide excreted in the urine were similar both in the presence and in the absence of quinidine or temsirolimus (Table 1). Statistical tests confirmed that the 90 % CIs for the ratio of geometric means between lenalidomide alone and lenalidomide plus an interacting drug were completely contained within the limits of 80 and 125 % for renal clearance of lenalidomide.

### Effect of lenalidomide on pharmacokinetics of co-administered drugs in plasma or whole blood

Key pharmacokinetic parameters of digoxin, temsirolimus, and sirolimus are summarized in Table 2.

**Table 2:** Pharmacokinetic parameters of digoxin, temsirolimus, and sirolimus in the absence and presence of lenalidomide

Pharmacokinetic Parameter	Digoxin (0.5 mg)		Temsirolimus (25 mg)		Sirolimus	
	Alone (*n* = 17)	+Lenalidomide (*n* = 17)	Alone (*n* = 14)	+Lenalidomide (*n* = 11)	Temsirolimus alone (*n* = 13–14)	+Lenalidomide (*n* = 11)
AUC_t_ (h ng/mL)	19.1 (30.9)	20.4 (27.8)	2,666 (27.7)	2,573 (25.6)	4,583 (21.9)	4,576 (22.3)
AUC_∞_ (h ng/mL)	26.3 (29.0)	28.1 (23.8)	2,804 (25.8)	2,713 (22.9)	5,357 (22.1)	5,371 (23.8)
*C*_max_ (ng/mL)	1.69 (37.6)	1.93 (33.0)	586 (35.1)	649 (19.6)	54.0 (29.0)	56.2 (30.5)
*T*_max_ (h)	1.0 (1–3)	1.0 (1–3)	0.47 (0.25–2)	0.47 (0.25–0.5)	1.5 (1–36)	1.5 (1–24)
*t*_1/2_ (h)	32.3 (35.0)	36.8 (27.6)	15.3 (28.5)	15.2 (27.2)	59.9 (13.7)	61.2 (9.9)

Geometric mean (geometric CV %) data are presented for all parameters except for *T*_max_ where median (range) data are presented

*AUC* area under the plasma concentration curve, *AUC*_*t*_ AUC from time zero to the last measurable concentration, *AUC*_*∞*_ AUC from time zero to infinity, *CL*_*R*_ renal clearance, *C*_*max*_ maximum observed plasma concentration; fe, cumulative urinary excretion as a percentage of administered dose, *ND* not determined, *t*_*1/2*_ z terminal-phase half-life, *T*_*max*_ time to reach *C*_max_

**Fig. 4:**
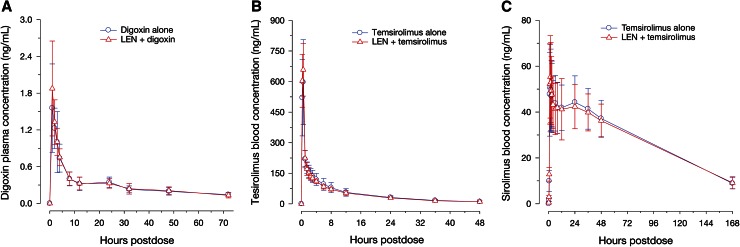
Mean (standard deviation) plasma concentration–time profile of (**a**) digoxin, (**b**) temsirolimus, and (**c**) sirolimus in the absence and presence of lenalidoimide

Examination of the digoxin concentration–time profiles showed that except for the small difference in the peak concentration (at 1-h post dose), the curves for lenalidomide co-administration versus placebo co-administration were almost superimposed, suggesting lenalidomide does not affect digoxin distribution and elimination (Fig. 4a). The observed median digoxin *T*_max_ was identical when co-administered with either lenalidomide or placebo (1 h; range 1–3 h). The 90 % CI for the ratio of geometric means between digoxin plus placebo and digoxin plus lenalidomide for AUC_t_ (Fig. 2a) was completely contained within the limits of 80 and 125 %. The mean C_max_ value of digoxin was approximately 14 % higher in a statistically significant manner (i.e., the upper limit of the 95 % Cl exceeded 125 %) when co-administered with lenalidomide versus placebo (Fig. 2b).

The mean plasma concentration–time profiles of temsirolimus (Fig. 4b) or sirolimus (Fig. 4c) were almost identical when temsirolimus was administered alone or co-administered with lenalidomide. There was no difference in *T*_max_ and terminal t_1/2_ of temsirolimus or sirolimus between treatments (Table 1). The 90 % CIs for the ratio of geometric means between temsirolimus alone, and temsirolimus plus lenalidomide were completely contained within the limits l of 80 and 125 % for *C*_max_ and AUC_t_ of temsirolimus (Fig. 2b).

### Quinidine concentrations in plasma

Visual inspection of the mean concentration–time profile suggests that the quinidine concentration approached steady-state on Day 4 and the mean trough concentration ranged from 1,705 to 1,844 ng/mL on Days 4–5. The mean *C*_max_ [percent coefficient of variation (CV %)] was 2,352 ng/mL (24.3 %) on Day 4.

### Safety

No remarkable safety findings were observed in these studies. There were no deaths or serious AEs. Of all reported AEs, 2 were moderate in severity (toothache and furuncle), and all others were mild in severity. The majority of the AEs were unrelated to study drug. There was no apparent increase in the number of subjects reporting AEs, or the number of AEs reported, when lenalidomide was given with digoxin, quinidine, or temsirolimus when compared with administration of lenalidomide alone or with placebo.

There were no clinically significant laboratory abnormalities or any apparent differences in the laboratory test results between study drug alone (lenalidomide, digoxin, quinidine, or temsirolimus) and the study drug co-administered with a tested interacting drug. Consistent with the known QT prolongation effect of quinidine, there was a trend toward increasing QT interval values corrected for heart rate (QTc) during treatment with quinidine. No difference was observed in vital signs between treatments.

## Discussion

The results presented here show that co-administration of lenalidomide with a P-gp substrate or inhibitor has no clinically significant effect on the pharmacokinetic disposition of either lenalidomide or the co-administered drugs. These results are consistent with in vitro findings demonstrating that lenalidomide has a very low affinity for P-gp as a substrate and is not a P-gp inhibitor.

Quinidine is a well-characterized, strong P-gp inhibitor often used to determine if a drug is a substrate of P-gp in vivo. Quinidine has a high in vivo inhibition potential on P-gp, as suggested by a value of approximately 1 for the ratio of the free-drug *C*_max_ in vivo to the inhibition constant (Ki) in vitro, which is higher than most well-known, “strong” P-gp inhibitors such as verapamil, itraconazole, ketoconazole, cyclosporine, and clarithromycin [17]. Furthermore, among several assessed P-gp inhibitors, quinidine causes the largest increase in digoxin AUC and *C*_max_ in clinical studies [12]. Quinidine has also been proven to significantly reduce renal clearance of digoxin in humans [18, 19]. Thus, quinidine was selected to maximize the likelihood of detecting a P-gp mediated drug-interaction with lenalidomide. Using quinidine as the probe drug, the data confirms that P-gp does not play a role in the pharmacokinetic disposition of lenalidomide. The observed mean quinidine trough concentration (1,705 ng/mL) was comparable to the historical concentration (1,430 ng/mL) reported for significantly inhibiting renal clearance and increasing plasma AUC of digoxin in healthy subjects [18]. In addition, the estimated mean *C*_max_ for unbound quinidine (approximately 1 μM, assuming 15 % of unbound drug) in our study, was close to the in vitro Ki (0.9 μM) reported for inhibiting human P-gp transporters in vitro [17]. Thus, plasma quinidine exposure was adequate for inhibition of P-gp. Lenalidomide mean renal clearance and total urinary excretion (as percentage of administered dose) were comparable when lenalidomide was administered alone or in combination with quinidine, demonstrating that the rate and capacity of lenalidomide renal excretion were not affected by P-gp inhibition. Similarly, the median *T*_max_ and the oral bioavailability (as indicated by percentage of dose excreted in urine) of lenalidomide remained unchanged when lenalidomide was co-administered with quinidine, suggesting that the rate and extent of lenalidomide oral absorption were also not altered by P-gp inhibition. Consequently, there was no clinically relevant change in the plasma exposure to lenalidomide when co-administered with quinidine.

Given the lack of effect by quinidine on lenalidomide exposure, it is unlikely that co-administration of other commonly used P-gp inhibitors or substrates would cause a clinically significant increase in the systemic exposure to lenalidomide, as confirmed in our studies using digoxin or temsirolimus.

Digoxin is recommended by the US Food and Drug Administration as a P-gp substrate probe to determine whether a drug is an inhibitor of P-gp. Like lenalidomide, digoxin undergoes limited metabolism and is eliminated predominately through renal excretion of the unchanged drug. Due to this absence of metabolic clearance, digoxin pharmacokinetics shows the highest perturbation with effective P-gp inhibitors in vivo as reported previously [12]. However, except for a 14 % increase in *C*_max_, no other meaningful changes in digoxin pharmacokinetic were observed after multiple doses of lenalidomide.

Although it was statistically significant, a 14 % increase in *C*_max_ of digoxin is unlikely to be clinically relevant. The underlying mechanism for this minor increase in *C*_max_ cannot be explained by direct inhibition of P-gp because lenalidomide does not inhibit digoxin transport by P-gp in vitro. To date, there has been no evidence in the literature of any significant toxicity due to concomitant use of lenalidomide and digoxin. However, because digoxin has a narrow therapeutic window and repeated doses of lenalidomide are known to cause hematological toxicities, a lower dose of lenalidomide (10 mg/day) was administered in our study to minimize any potential toxicities. Periodic monitoring of digoxin concentration is still recommended during lenalidomide therapy.

As with quinidine or digoxin, the present studies further demonstrate that there are no meaningful pharmacokinetic interactions between lenalidomide and temsirolimus. Both temsirolimus and its active metabolite sirolimus are substrates and inhibitors of P-gp in vitro. However, the role of P-gp in temsirolimus deposition in vivo is considered to be limited, because temsirolimus is given intravenously and excreted minimally in urine, thereby excluding the involvement of intestinal and renal P-gp. Pharmacokinetic drug interactions due to P-gp inhibition are also not expected between temsirolimus and lenalidomide, as lenalidomide is not a P-gp inhibitor in vitro [14, 20]. In addition, lenalidomide would not affect metabolism of temsirolimus via CYP3A4, which is the main elimination pathway of temsirolimus, because lenalidomide is not a substrate, inhibitor, or inducer of CYP3A4 [21]. Thus, it is intriguing that an increase in the exposure of both drugs was observed when lenalidomide was co-administered with temsirolimus in an uncontrolled phase I study [15]. In well-controlled settings, our findings are consistent with the known mechanisms underlying pharmacokinetic disposition of lenalidomide and temsirolimus and the in vitro and in vivo correlation prediction.

The conflicting outcomes noted between the results described here and the studies previously reported in the literature highlight the importance of evaluating drug–drug interactions under well-controlled conditions. Both studies were conducted in MM patients taking multiple co-medications, and with multiple comorbidities [15, 16]. In addition, as lenalidomide elimination is highly dependent upon renal function and temsirolimus elimination is highly dependent upon hepatic metabolism, the contribution of imbalanced renal or hepatic function between treatment groups to pharmacokinetic variability should not be overlooked. Finally, the reported phase I study in MM patients did not collect lenalidomide or temsirolimus data under monotherapy and thus lacked a control group for comparison with the combination therapy [15]. In contrast, our studies were conducted in healthy subjects with a crossover design, which not only controls for co-medications and organ function but also allows each subject to serve as their own control, thereby reducing the impact of the inter-individual variability.

It is possible that changes induced by MM in the proximal tubules of the kidneys (i.e., toxic effect of monoclonal light chain deposition) could alter the role of P-gp in lenalidomide clearance, thereby leading to clinically significant drug interactions that are not seen in healthy volunteers. However, we have not found any obvious difference in lenalidomide clearance between MM patients and other study populations, arguing against this possibility [9, 22].

In conclusion, co-administration with substrates or inhibitors of P-gp had no clinically relevant effect on the pharmacokinetics of lenalidomide. Similarly, coadministration with lenalidomide had no clinically relevant effect on the pharmacokinetics of temsirolimus. Although there was a minor increase in digoxin *C*_max_ when co-administered with lenalidomide, no other changes in digoxin pharmacokinetics were observed upon the co-administration. Based on these results, lenalidomide can be co-administered with a P-gp inhibitor or substrate with little or no interaction expected.
